# Single chain fragment variable, a new theranostic approach for cardiovascular diseases

**DOI:** 10.3389/fimmu.2024.1443290

**Published:** 2024-12-13

**Authors:** Rukhshan Zahid, Juncheng Wang, Zecheng Cai, Ayesha Ishtiaq, Meng Liu, Dan Ma, Yan Liang, Yuekang Xu

**Affiliations:** ^1^ Anhui Provincial Key Laboratory for Conservation and Exploitation of Biological Resources, College of Life Science, Anhui Normal University, Wuhu, Anhui, China; ^2^ College of Life Science, Anhui Normal University, Wuhu, Anhui, China

**Keywords:** cardiovascular diseases, single-chain fragment variable, diagnostics, therapeutics, antibody engineering

## Abstract

Cardiovascular diseases (CVDs) remain a significant global health challenge, leading to substantial morbidity and mortality. Despite recent advancements in CVD management, pharmaceutical treatments often suffer from poor pharmacokinetics and high toxicity. With the rapid progress of modern molecular biology and immunology, however, single-chain fragment variable (scFv) molecule engineering has emerged as a promising theranostic tool to offer specificity and versatility in targeting CVD-related antigens. To represent the latest development on the potential of scFv in the context of CVDs, this review summarized the new mechanism of action and applications as therapeutic, as well as diagnostic agents. Furthermore, the advantages of scFv, including its small size, ease of modification, and ability to be engineered for enhanced affinity and specificity, are also described. Finally, such challenges as immunogenicity, stability, and scalability, alongside strategies to overcome these hurdles, are deeply scrutinized to provide safer and more effective strategies for the diagnosis and treatment of the incurable CVDs.

## Introduction

Being the leading cause of death in the world today, cardiovascular diseases (CVDs) comprise conditions like heart failure, arrhythmias, atherosclerosis, coronary heart disease, myocardial infarction, peripheral arterial disease, deep vein thrombosis, and inflammatory heart disease. According to a survey by the World Heart Federation, CVDs are currently estimated to cause 20.5 million deaths each year (1). In the coming decade, however, the mortality rates associated with CVDs are expected to rise due to a considerable increase in risk factors, such as obesity, elevated cholesterol, diabetes mellitus, hypertension, smoking, a sedentary lifestyle, poor nutrition, and aging population (2), as most CVD cases and deaths are primarily driven by modifiable risk factors, some of which have global implications, while others differ based on economic conditions, indicating the need for targeted health policies (3). Approximately 55 million deaths were estimated worldwide in 2017, with 17.7 million attributed to cardiovascular diseases (4). Alarmingly, projections indicate that CVD deaths are predicted to increase to over 23.6 million by 2030 (5).

Atherosclerosis is the main trigger factor for the majority of CVDs and goes through a process of development as it advances. The endothelial dysfunction associated with many internal and external influences can increase the permeability of capillary walls to macromolecules. The oxidized low-density lipoprotein (ox-LDL) and the oxidized phospholipids (OxPLs) on low-density lipoprotein (LDL) are modifications caused by enzymes and reactive oxygen species. These modifications increase the likelihood of LDL crossing vascular walls or accumulating nearby (6–8). OxPLs can bind to a wide variety of receptors on immune cells (such as CD36 or LOX-1), leading to initiate an expression of cytokines, chemokines, matrix metalloproteinases (MMPs), and adhesion molecules that exacerbate the situation of plaque inflammation and instability (8). Ox-LDL metabolite activates platelets and induces thrombosis by promoting the expression of tissue factors and inflammatory mediators (9). In the meantime, dysfunctional vascular endothelial cells can also cause elevated levels of inflammatory factors like monocyte chemotactic protein-1, which leads to the recruitment of monocytes toward the endothelial cells. Monocytes, once at the endothelium, are transformed into macrophages in response to the action of the adhesion molecules like vascular endothelial adhesion molecule (VCAM-1) and intercellular adhesion molecule-1 (ICAM-1) (10). The monocyte-derived macrophages then become the main actors in the whole atherosclerosis and can be presented as different phenotypes and perform distinct functions depending on the microenvironment condition. When macrophages recognize and absorb ox-LDL, these cells are converted into foam cells (11). For the second stage, vascular smooth muscle cells (VSMCs) are the main players and undergo the process of transitioning from the epitome of a contractile phenotype to a synthetic one due to the influence of immune cells and inflammatory factors. These VSMCs migrate from the middle layer of the arterial walls to the intima, where they begin to proliferate. Some VSMCs phagocytose ox-LDL and convert to VSMC-derived foam cells, while others synthesize extracellular matrix molecules (like collagen) to form fibrous caps. The new intima is formed through this process, and the vascular remodeling occurs. After that, the foam cells gradually undergo necrosis or apoptosis, contributing to the formation of necrotic cores. This leads to either thrombosis or plaque rupture. At the stage of necrotic core rupture, synthetic VSMCs are known to secrete MMPs, which degrade collagen and weaken the fibrous cap. In addition, the necrotic core is also initiated by prolonged oxidative stress (12–15). However, the expansion of necrotic cores, as well as the development of new vessel growth, can result in plaque rupture and the formation of thrombosis. Therefore, persistent arterial spasms eventually lead to critical lumen occlusions (14). Myocardial infarction leads to the death of numerous cardiomyocytes due to impaired energy metabolism, even with prompt blood reperfusion. Additionally, adverse reactions such as the substantial buildup of oxygen free radicals due to ischemia and hypoxia, calcium ion (Ca2+) overload, and inflammatory cascade reactions in myocardial cells can exacerbate mitochondrial dysfunction and myocardial injury. This may result in severe complications such as malignant arrhythmias, myocardial fibrosis, or heart failure (16).

The atherosclerosis-related CVDs are treated by different approaches depending on each patient’s risk stratification and disease severity. The main objective of all treatment plans for CVD is to enhance blood circulation while limiting the extent of tissue damage, thereby preserving the loss of cardiomyocytes and improving their contractile function. More advanced cases of CVD would necessitate surgical procedures like clot removal, implantation of artificial cardiac pacemakers for arrhythmias, and physical correction of pathological changes in the heart, which make the operation very complicated and sometimes very dangerous. On the other hand, sticking to regular drug therapy is also challenging; the treatment is usually lifelong (17). For example, statins are drugs that lower cholesterol levels and reduce the risk of CVD events and death. Statins work by blocking a step in cholesterol production in the liver. Statins can also slow down the growth and change the composition of atherosclerotic plaques, which are the main cause of CVD (18). Aspirin is the most used drug for the secondary prevention of CVDs (19). β-blockers are drugs that inhibit the action of catecholamines on adrenergic receptors and thus reduce sympathetic stimulation and cardiotoxic effects. Therefore, β-blockers are also recommended as the initial therapy for atrial fibrillation and CVDs; however, they are contraindicated for patients with hypertension (20). Angiotensin-converting enzyme inhibitors and angiotensin II receptor blockers are the preferred pharmacological agents for managing heart failure, coronary artery disease, MI, and hypertension (21). The renin-angiotensin-aldosterone system (RAAS) helps to regulate blood pressure and fluid balance. In heart failure, it can become overactive, leading to higher blood pressure and fluid buildup. By blocking its activation, this medication helps relax blood vessels and reduce fluid, easing the strain on the heart. This medication inhibits an enzyme called enkephalinase, which breaks down enkephalins, natural substances in the body that act like painkillers. By preventing their breakdown, enkephalin levels rise, potentially contributing to pain relief and improved well-being for patients with heart failure (22). The Prospective comparison of ARNI with ACEI to Determine Impact on Global Mortality and morbidity in Heart Failure (PARADIGM-HF) and Prospective comparison of ARNI with ARB Global Outcomes in HF with preserved ejection fraction (PARAGON-HF) trials have confirmed that sacubitril valsartan has an established role in the treatment of patients with heart failure with reduced or preserved ejection fraction (23, 24). Although significant progress in existing treatments has been made in the past decade, the therapeutic effects of pharmacotherapy are suboptimal because of the non-specific cytotoxicity, poor solubility and absorption, first-pass metabolism, poor biocompatibility, and low bioavailability of existing cardiovascular drugs (25).

The recent achievements in molecular and structural biology, together with a better understanding of the immune system and its capabilities in fighting infection and disease, have opened novel horizons for modifying immune cell behavior to improve and, possibly extend the human lifespan (26). The discovery of hybridoma technology in the 1970s was a significant breakthrough, paving the way for the creation of the first monoclonal antibody (mAb) therapeutic. Orthoclone OKT3 developed by Janssen-Cilag, was the first FDA-approved mAb therapy, that targeted T lymphocytes and was used to prevent organ rejection in transplant patients (27, 28). These technologies kick-started a new drug modality—biologics—with ∼160 protein therapeutics approved for clinical use to date (29). Biologics like (but not exclusive to) immunoglobulins such as mAbs are currently the drug modality most pursued in therapeutic development (30). However, the limitations around single targeting using mAbs and rising unmet medical needs inspired researchers to go further and conceptualize molecules with multispecificity (31). To circumvent the Fc-associated effects and the large size of IgGs in specific clinical scenarios, smaller antibody molecules such as fragments of antigen-binding (Fab) and single-chain variable fragments (scFv) are gaining traction as both therapeutics and diagnostics (32). In a review, Muñoz-López et al. discussed the potential of scFv in cancer diagnosis and therapy. They highlighted the high specificity and affinity of scFv for antigens, low immunogenicity, and the ability to penetrate tumor tissues (33). The versatility of scFv fragments extends across a broad spectrum of applications. Being an invaluable molecular instrument for altering protein activity within living organisms (34). scFv fragments have many applications, such as molecular tools to change protein functions *in vivo*, delivery agents of radionuclides in diagnostic imaging (35, 36), and possible therapeutics for many diseases, such as cancer, HIV, neurodegenerative diseases, and atherosclerosis (37–40). This shift is evidenced by the approval of several Fab and scFv format antibodies, including Abciximab, Ranibizumab, Certolizumab pegol, and Idarucizumab in the Fab format, as well as Blinatumomab and Brolucizumab in the scFv format, marking a significant advancement in the field of biologic therapeutics (41). Furthermore, scFvs can be used for the targeted delivery of imaging agents for diagnostic purposes, or drugs and cells to specific sites for therapeutic gains (42), in various CVDs, including myocardial ischemic reperfusion injury (IR) (43), thrombosis (44) and atherosclerosis (40). In this review, we review the theranostic (diagnostic and therapeutic) development of scFv in the field of CVDs and discuss the limitations of scFv at the clinical levels. More importantly, scFv for atherosclerosis accompanied by its complications such as arrhythmia, ventricular remodeling, MI, thrombosis, and myocardial IR are referred since they are crucial diseases among CVDs.

### Single-chain fragment variables

**Figure 1:**
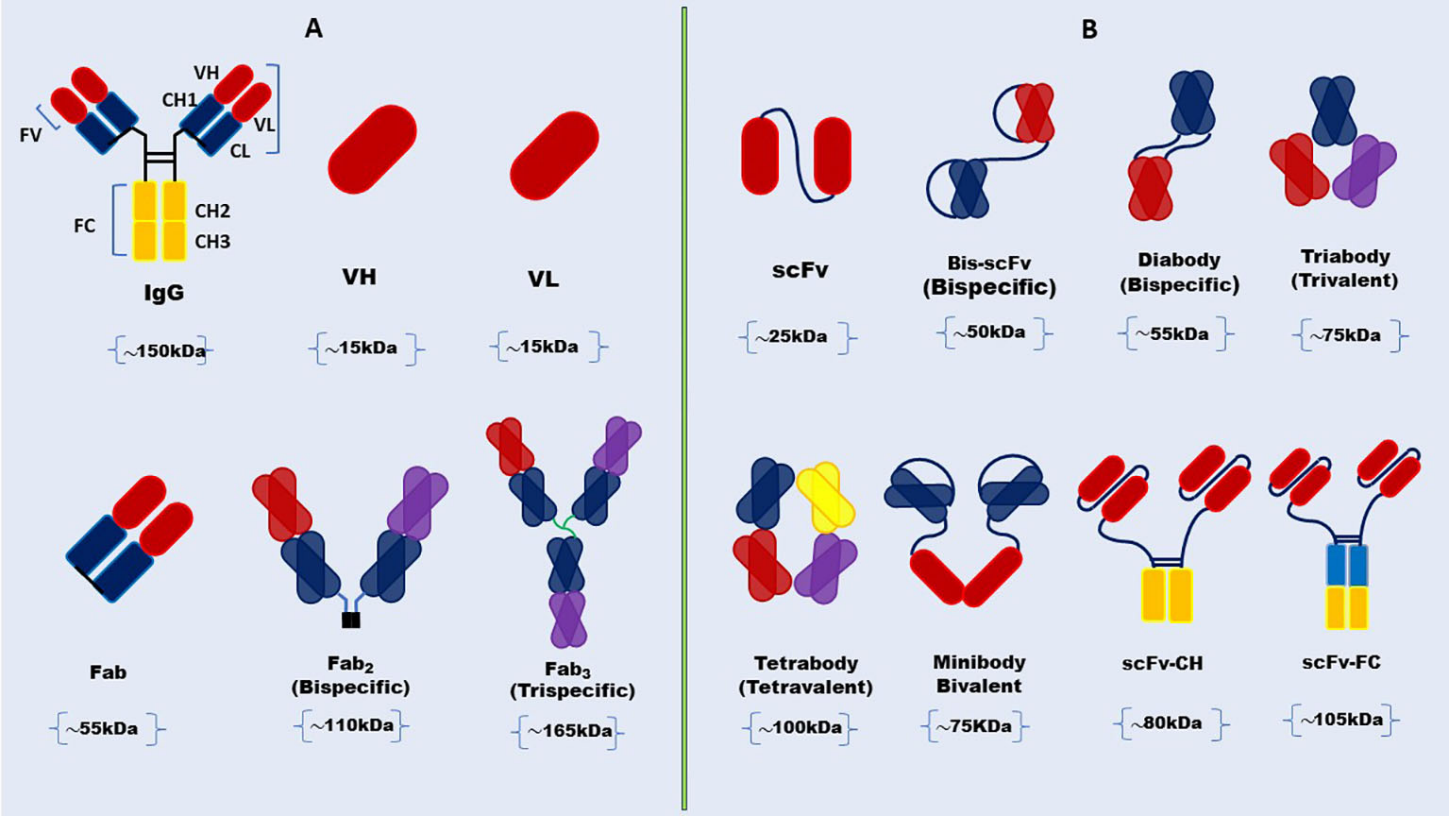
Schematic representation of full-length antibody and structural diversity of antibody Fab fragments and scFv formats. **(A)** Full-length antibody structure showcases the heavy and light chains, the variable (V), and constant regions with structural diversity of antibody Fab fragments. **(B)** scFv and its various engineered formats, including diabody, triabody, tetrabody, minibody, and scFv-Fc.

Antibodies are proteins that protect us from foreign invaders like bacteria and viruses. They have antigen-binding sites, called paratopes (like locks), at the ends of their “Y” shaped structures. These paratopes match one specific epitope (like a key) on the antigen and bind to it tightly (45). Antibodies neutralize foreign bodies by recognizing and binding a specific epitope on an antigen through the two fragments of antigen-binding sites (Fab). The constant fragment (Fc) domain recruits cytotoxic effector molecules by binding to Fc receptors and interacting with the neonatal Fc receptor, which also gives long serum half-lives (46). Full-length mAbs have been used as tools for various therapeutic applications (47). However, the Fc-mediated effect can be harmful in some situations. For example, it can cause toxicity by activating Fc receptor-expressing cells and releasing cytokines. The long serum half-life is also a drawback in imaging applications, where quick clearance is considered important in reducing exposure to radionuclide molecules (48). Consequently, mAbs have been modified to prevent these shortcomings and maximize their therapeutic and diagnostic performance. The initial step was the cutting of the Fc domain by enzymes, such as papain and pepsin (49), leaving only Fab fragments. Then, antibody engineering helped to reduce the size of Fab further to tiny fragment variables (Fv), monovalent Fabs, Fab_2,_ diabody, triabody, tetrabody, as well as minibodies (**Figure 1**) (32, 33, 50–53). These fragments have been modified to improve the binding affinity, pharmacokinetics *in vivo*, stability, and expression levels of antibodies (54). scFvs have been taken as an alternative to full-length mAb and other antibody fragments in diagnostic and therapeutic applications and made up 35% of antibody fragments in clinical trials based on the data up to 2010 (33, 55).

Fv is the smallest part of an immunoglobulin molecule that can bind to antigens. A single chain of the Fv (~25 kDa) is a scFv fragment, which has variable heavy (VH) and variable light (VL) chains connected by a flexible peptide linker. The first scFvs were cloned in 1988 (52, 56). This scFv was made functional in *E. coli*, indicating human success in changing the features of scFv by protein engineering for enhanced affinity and modified specificity (57). The linker’s length and amino acid composition are essential for the proper folding of these proteins. They need to have a hydrophilic sequence to prevent the peptide from inserting into or between the variable domains during the protein folding (58, 59). The flexible DNA linker that joins the two variable (V) domains is crucial for the proper folding of the polypeptide chain. It has been estimated before that the peptide linker should cover 3.5 nm (35 A) between the carboxy end of the variable domain and the amino end of the other domain without impairing the folding and formation of a complete antigen-binding site (60). Besides the linker peptides created from scratch, peptide sequences based on known protein structure have been used to give a suitable length and shape to link the variable domains of a scFv without significant steric hindrance (61). The most widely used designs have sequences with glycine and serine residues for flexibility and charged residues like glutamate and lysine to improve solubility (62–64). Upon the successful linkage of the variable chains in scFvs via a peptide linker, bacterial cells are predominantly employed as the expression system, although alternative platforms such as mammalian cells, yeast, plants, and insect cells are also utilized (65).

## Types of scFv, delivery routine, and improvement of efficacy

Genetic engineering advancements have led to the creation of various types of scFv with different formats and mechanisms of action. In spite of a modern biological product, the diminutive size of scFvs also results in a shorter bloodstream half-life, necessitating the development of more elaborate structures to deliver lasting therapeutic and diagnostic benefits (34, 66). These compact antibody formats retain antigen recognition ability but lack the Fc region, giving rise to both simple antibodies and intricate molecules like bi-specific, tri-specific, tetra-specific antibodies, and scFv-Fc (67) shown in **Table 1**.

**Table 1:** Comparison between scFv and other antibody fragments.

Antibody fragments	Molecular weight (kDa)	Characteristics	Advantages	Disadvantages	References
scFv	~25-30	Single-chain variable fragment; VH and VL domains linked by a peptide	Small size, Low molecular weight, high specificity, easy production and modification, low immunogenicity, ease of genetic manipulation, and good tissue penetration	short half-life, rapid clearance, and Low avidity	(59)
Bispecific antibody	~50	Two distinct antigen-binding domains linked together	Can bind with two targets	Have lower affinity than full antibodies, Stability issues, production challenges, and potential for increased immunogenicity	(33, 68)
Diabody	~55	Non-covalently linked dimer of two scFvs	Higher avidity, Enhanced binding	Potential for immunogenicity, Complex production, potential for aggregation	(34, 69–71)
Triabody	~75	Three scFvs or Fab fragments connected by linkers	Increased binding to multivalent antigens and enhanced therapeutic potential	Complex structure may affect stability, High production complexity, potential immunogenicity	(33, 69, 71)
Tetrabody	~100	Four scFvs linked together	High avidity, can cross-link multiple targets	More complex to produce, potential for aggregation and stability issues	(71)
Minibody	~75	scFv linked to a dimerization domain (e.g., CH3 domain of IgG)	Intermediate-size, better stability, and longer half-life than scFv	Reduced affinity and larger size than scFv, may limit tissue penetration	(34, 72)
scFv-Fc	~105	scFv fused to Fc region of IgG	Improved half-life, Fc-mediated effector functions	Larger size may limit tissue penetration and be more complex to produce and has the potential for increased immunogenicity	(73)
Fab	~50-55	Fragment antigen-binding; contains one constant and one variable domain	Moderate molecular weight, high specificity, easy production and modification, low immunogenicity, better tissue penetration than IgG	Low stability, aggregation tendency, short half-life, low avidity, and more complex to produce	(34, 74)

scFv were traditionally made from hybridoma cells in immunized animals by amplifying the VH and VL domains from the mRNAs of responding antibodies, and connecting them with a polylinker before they can be put in a vector of choice (75). To overcome the limitations of conformation restriction, display technologies (phage, yeast) *in vitro* lately have replaced hybridoma technology because they be changed to improve scFv properties and make a group of very diverse and highly functional antibodies (76). Bypassing the need to get antibodies from the immunized host, synthetic antibody libraries allow the introduction of extreme diversity in the scFvs, as they change the complementarity-determining regions (CDR) regions through synthetic DNA (76).

**Figure 2:**
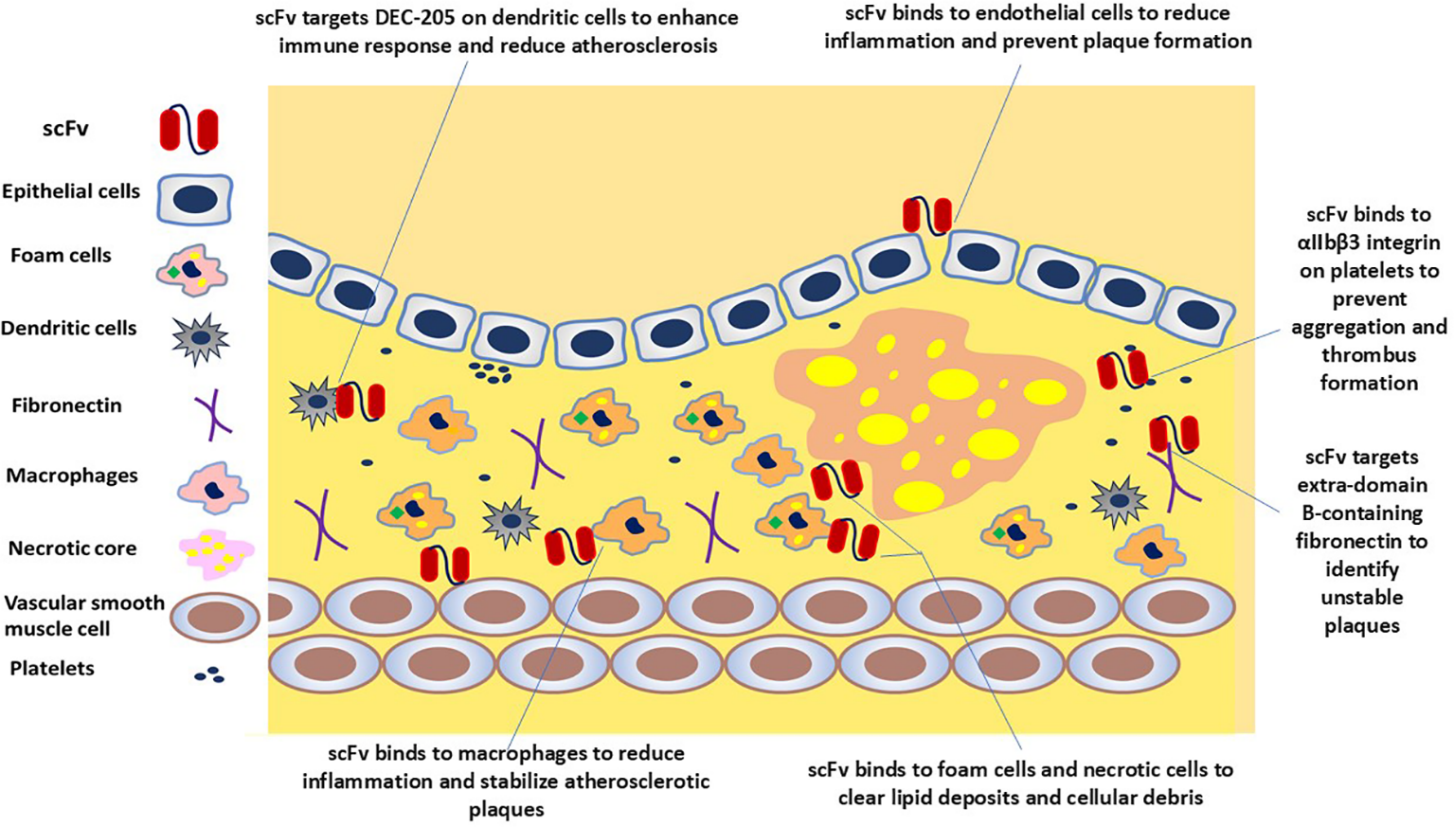
scFv-based strategies to target atherosclerotic plaque. scFv molecules could potentially bind to endothelial cells, macrophages, dendritic cells, foam cells, necrotic cells, and platelets, illustrating the ameliorative potential of scFv to neutralize targets and mitigate the progression of atherosclerosis.

**Figure 3:**
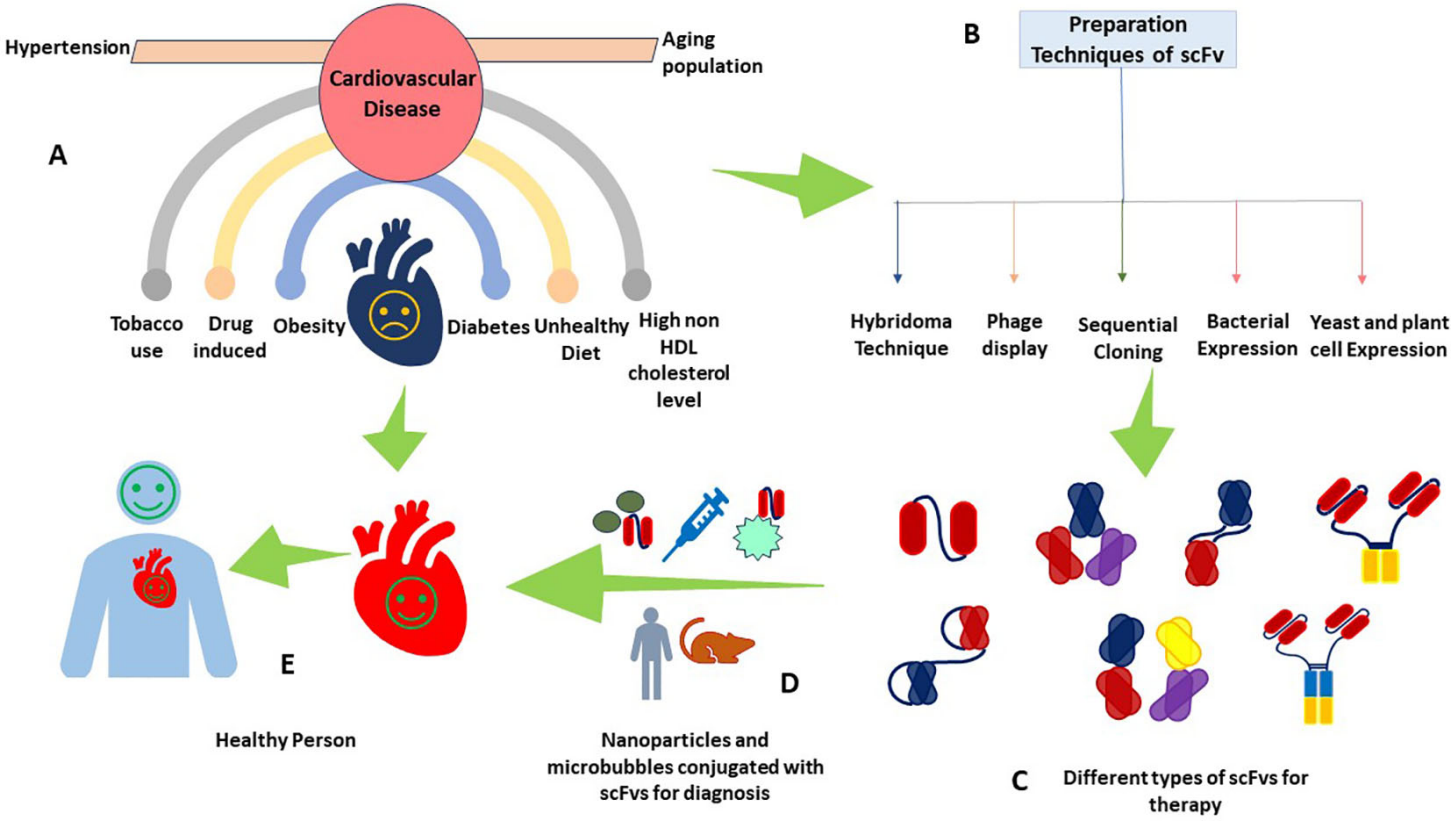
scFv as a theranostic agent in CVDs. This figure provides a multifaceted view of CVDs depicting **(A)** common risk factors contributing to CVDs, including obesity, diabetes, smoking, and drug-induced hypertension, **(B)** the different production techniques of scFv antibodies, **(C)** different types of scFv antibodies for therapeutics, **(D)** the cutting-edge integration of scFv with nanoparticles and microbubbles, a leap forward in diagnostic methodologies, and **(E)** a healthy individual with a healthy heart, a testament to the successful application of scFv-based theranostics.

For the *in vivo* applications and administration route of an appropriate scFv, delivery vehicle, concentration maintenance, and toxicity effects are important, which can be administered systemically when needed or overexpressed *in vivo* using viral vectors (34). The route of scFv is selected based on the required pharmacokinetics, pharmacodynamics, and biodistribution. Through intravenous administration, systemic distribution and arterial wall penetration are ensured. Intramuscular or intranasal ways get a localized expression and a secretory function by transduced cells. For targeted delivery of specific sites, intracranial or intraperitoneal administration is performed (41, 77). These delivery modes are helpful when long-term expression is not needed, for example, in imaging applications. However, in situations where a constant level of antibody is required, viral-mediated delivery is a good option (34). One of the common ways to deliver scFv fragments is by using adeno-associated virus (AAV) vectors and *in vivo* phage display because vectors are non-pathogenic, non-toxic, and have low immunogenicity, which means they do not cause disease, harm, or immune response in the host (78, 79). Moreover, the affinity, specificity, avidity, and valency of this scFv will determine the targeting efficiency, accessibility, and turnover of the target antigen or receptor (41). scFvs are small and have high penetration so they can get into tumors better, but they also leave the blood faster. Therefore, some studies discuss different ways to make scFvs last longer, like adding polyethylene glycol (PEG) or albumin (34, 48). In particular, conjugation with PEG enhances scFv’s hydrophilicity, stability, circulation time, and biodistribution, making scFv more versatile in therapeutic and diagnostic applications (80). For targeted therapy, scFv can be coupled with drugs (81), and toxins (82), to direct these agents to specific cells or molecules within plaques such as dendritic cells, fibronectin, necrotic core, and platelets (**Figure 2**) (17). Imaging abilities are enhanced by tagging scFv with radionuclides, fluorescent dyes, or magnetic nanoparticles, which can be visualized as Positron emission tomography (PET), fluorescence, and magnetic resonance imaging (MRI) imaging modalities for diagnostic purposes (**Figure 3**) (11, 40, 83). When scFvs are attached to radionuclides in radiotherapy applications, their faster rate of removal from blood reduces exposure to healthy tissue (36). These fragments can be engineered to connect to different moieties, such as drugs, toxins, radionuclides, quantum dots, or liposomes (34, 48, 82, 84). As an alternative to mAbs and other antibody fragments, scFvs are increasingly used in diagnostic and therapeutic applications due to their specific binding affinity to antigens, superior biodistribution, low immunogenicity (particularly when derived from human sources or engineered to minimize immune responses), low cost, and high modifiability (59, 85–89).

## Theranostic application of scFv for CVDS

### scFv as diagnostic agent

The advent of scFv has opened new frontiers in targeted diagnosis. Conjugation of scFv antibodies with imaging agents enables them to be trackable via MRI modalities, magnetic particle imaging (MPI), near-infrared fluorescence (NIR) imaging, and PET (11, 40, 83). For example, ASA6, a scFv antibody linked to NaNdF4@NaGdF4 nanoparticles, allows noninvasive visualization of atherosclerotic plaques by MRI and NIR-II imaging techniques (11). Likewise, MRI and NIR techniques were utilized to effectively demonstrate the potential of multimodal imaging nanoparticles in enhancing imaging efficacy. These nanoparticles were functionalized with anti-αIIbβ3 scFv fragments of the TEG4 antibody, to improve the accuracy of atheroma plaque detection in the ApoE−/− mouse model, which preserved the activity and avidity of the TEG4 antibody, for specific targeting of activated platelets within the plaques, and employed both *in vivo* and *ex vivo* imaging for robust diagnostic capabilities (90). Furthermore, a study developed oil-in-water nano-emulsions (NEs) loaded with superparamagnetic iron oxide (SPIO) nanoparticles for MRI. These NE-SPIO nanoparticles were coated with a PEG layer, which was functionalized with a fully human scFv-Fc antibody (P3) that specifically recognizes galectin 3, an atherosclerosis biomarker. Immunohistochemistry analysis confirmed that the P3-functionalized formulation effectively targeted atheromatous plaques in mouse aorta and human artery sections for diagnosis. This study assesses various NE formulations, and among them, NE-PEG3400-maleimide formulation (#3) exhibited the lowest liver uptake and best stealth properties, making it ideal for conjugation with the scFv-Fc-2Cys P3 HuAb to form NE-P3 (#5). This conjugation increased the NE diameter to 199.5 nm, maintaining a monodisperse profile, and the dynamic MRI evaluation of NE-P3’s *in vivo* clearance showed a half-life of 103 minutes, which suggested a potential for enhanced contrast in distinguishing atherosclerotic lesions from non-specific signals due to its optimized clearance profile (91). Prévot et al. also introduced NEs loaded with SPIO nanoparticles, biofunctionalized with the scFv-Fc TEG4-2C antibody, to target atheroma plaques. These NEs retained their size and charge, exhibited promising MPI and MRI signals, and selectively adhered to atheroma plaques in both *in vitro* and ex vivo models (83). In continuation of their previous investigation, the researchers used a baculovirus-insect cell system to purify and engineer the scFv-Fc TEG4-2C antibody, that binds to platelets within the atheroma plaque. Subsequently, the conjugation of atheroma-specific antibodies to NEs through a heterobifunctional linker ensued, leading to the successful labeling of atheroma in rabbit arterial sections for visualization of diseased tissues. Collectively, these findings underscored the potential of NEs as a promising tracer for atherosclerosis imaging in MPI and MRI (92). Moreover, scFv-conjugated iron oxide nanoparticles also showcased the enhanced sensitivity and accuracy of MRI precise visualization, early detection, and monitoring of cardiovascular conditions (42). In addition to MRI, NIR imaging has also emerged as a valuable tool for diagnosis. For instance, the use of anti-extra-domain B-containing fibronectin (ED-B) scFv, conjugated with the NIR fluorescing/emitting tetra sulfonated carbocyanine dye for NIR imaging, revealed a significant correlation between the NIR-fluorescence uptake of aortas and the extent of the atherosclerotic lesions determined by Sudan staining. This heightened expression was linked with macrophage infiltration, suggesting potential plaque instability in ApoE-deficient mice (93). A novel PET imaging probe, 64Cu-3H3-scFv, demonstrated high specificity for β2-glycoprotein I (β2GPI) complexed with ox-LDL shown in **Table 2**. This probe achieved ideal blood clearance and distribution within 24 hours, significantly shorter than that of an intact IgG-based imaging probe, which helps to minimize background signals and enhance contrast between target atherosclerotic lesions and surrounding tissues. Additionally, the accumulation of 64Cu-3H3-scFv in aortic segments of WHHL rabbits was 2.8 times higher than that of controls, contributing to a higher target-to-background ratio and providing clearer, accurate imaging of atherosclerotic plaques (40).

**Table 2:** scFv-based theranostic that have been developed and tested to target specific molecules or cells in the atherosclerosis plaques.

Targets	Antigen/Receptor	Disease targeted	Model organism used	Clinical Application	References
Ox-LDL	β_2_GPI	Atherosclerosis	Rabbit	Diagnostic	(40)
Galectin-3	LOX-1	Atherosclerosis	Rabbit	Diagnostic	(79)
Platelets (LIBS-MBs)	GPII/GPIII	Thrombosis	Mouse	Diagnostic	(94)
Carbonic anhydrase II	Endothelial cell	Atherosclerosis	Rabbit	Diagnostic	(95)
Platelet and Stem cells	Sca1+GPIIb/IIIa	Ischemia	Mouse	Therapeutic	(43)
Macrophages	CD36 CD14	Atherosclerosis	Mouse	Therapeutic	(96)
OxPLs	Phosphocholine	Atherosclerosis	Mouse	Therapeutic	(97)
VCAM-1	Angiotensin II	Atherosclerosis	Mouse	Theranostic	(98)
Platelets	GPIIb/IIIa	Thrombosis	Mouse	Theranostic	(99)

In the context of microbubbles (MBs) as ultrasound agents, promising applications of scFvs are also developed. Ligand-induced binding sites microbubbles (LIBS-MBs) were developed to be conjugated with a single-chain antibody specific for activated glycoprotein IIb/IIIa (**Table 2**). These LIBS-MBs effectively adhered to thrombi and enabled enhanced ultrasound imaging which allowed real-time detection and visualization of thrombi *in vivo* in a mouse model, with potential clinical applications in humans after further testing for atherosclerosis (94). A similar study also utilized microbubbles in conjunction with an echo-enhancing feature and a thrombus-targeting device, incorporating an activated-platelet-specific single-chain antibody, for the detection of thrombi via ultrasound. This microbubbles-specific interaction with activated platelets enabled precise diagnosis and real-time monitoring of the size of the thrombus (99). MB-coated VCAM-1 specific scFv have been investigated for imaging vascular inflammatory responses associated with atherosclerosis and abdominal aortic aneurysm (AAA). Since VCAM-1 is expressed in inflamed endothelial cells, making it a valuable target for early diagnosis and risk stratification (98). For instance, VCAM-1 was targeted in a study by SPECT and fluorescence imaging using a 99mtechnetium and cyanine5 labeled scFv to detect vulnerable atherosclerotic plaques effectively. This method demonstrated a higher uptake of the tracer in the aortic regions of atherosclerotic animal models compared to controls. In fluorescence imaging, CY5-scFv-VCAM1 showed a stronger signal in the aorta of atherosclerotic mice, where SPECT imaging and autoradiography confirmed significant tracer uptake in the aortic arch and abdominal aorta of atherosclerotic animals. Autoradiographic analyses revealed a 4.45-fold higher uptake ratio in experimental animals compared to the controls. The researchers suggested that these findings indicate enhanced contrast for imaging atherosclerotic lesions; however, they do not provide evidence for an actual increase in accuracy. Incorporation of a control probe lacking targeting specificity for VCAM1, or other antibodies specific to such lesions, would be beneficial (100).

Apart from VCAM-1, another biomarker of atherosclerosis to be targeted by various diagnostic scFv is electronegative LDL (−), a physiologically modified LDL that exerts atherogenic effects in macrophages by promoting differentiation and inflammation (101). For instance, the green fluorescent protein (GFP)-scFv outlined the successful expression of a chimeric protein that specifically bind to LDL (−). This specificity was confirmed through ELISA and confocal microscopy. The protein’s capacity to be taken up by macrophages alongside LDL (−) suggested its utility in non-invasive diagnosis (102). Bender et al. developed an anti-LDL (−) scFv, functionalized with nanocapsules, and demonstrated specific molecular recognition of LDL (−) without reacting to native LDL, indicating that the active site of this antibody fragment was preserved. This specificity suggests the potential for these nanocapsules to be used as diagnostic tools for detecting atherosclerosis by targeting LDL (−) in biological samples (103).

*In vivo*, technologies have significantly advanced the discovery and optimization of antibodies for diagnosis. For example, Robert et al. used *in vivo* biopanning and high-throughput screening to isolate human scFv antibodies by targeting early atherosclerotic lesions for detection. Initially, they selected antibodies from rabbits on a cholesterol-rich diet, confirmed their specificity in ApoE-deficient mice, and ultimately selected two high-affinity scFv clones (104). Later, Deramchia et al. used *in vivo* phage display in hypercholesterolemic mice, to identify six scFv clones specific to atherosclerotic tissues (95). Building upon their prior research, they used sequence-based selection to isolate seven scFv antibodies binding to endothelial cells and inflamed intima in a rabbit atherosclerosis model for diagnostic purposes (105). Hemadou et al. further advanced the field by screening a human scFv phage library in an atherosclerotic rabbit model, identifying 209 specific clones, with 60% confirmed to react with rabbit atheromas (106). Along this line, they also developed human scFv-Fc (P3) clones, which exhibited significant cross-reactivity with atherosclerotic lesions across rabbit, mouse, and human tissues, specifically targeting galectin-3. Confirmation of its precise targeting of atherosclerotic plaques was achieved using NIR imaging in an Apoe−/− mouse model (79).

In addition to the diagnostic studies mentioned earlier, ongoing research endeavors have also aimed to target various other molecules and employ alternative techniques for diagnostics, like oxidized low-density lipoprotein receptor-1 (LOX-1), a biomarker highly expressed in atherosclerotic lesions, was targeted by designing scFvs fused with LOX-1-binding heptapeptides (LTPATAI, FQTPPQL, and LSIPPKA) at various regions. This antibody fused with N-terminal peptides showed enhanced LOX-1-binding activity without compromising stability, suggesting the potential for diagnosing atherosclerosis-related diseases (107). Another research group focused on scavenger receptor B1 (SR-B1), the primary receptor for high-density lipoprotein (HDL), responsible for cholesterol removal from HDL and its return to circulation. They developed a highly specific ScFv antibody, facilitating the detection and imaging of native SR-B1 in live cells. This advancement offered vital insights into the behavior and interactions of SR-B1 at the plasma membrane (108). To demonstrate the potential of such antibodies as diagnostic tools for evaluating the risk of CAD-related atherosclerosis, an anti-HDL scFv antibody was developed, which can distinguish between HDL from coronary artery disease (CAD) patients and healthy individuals (109). Similar to different molecules targeted, different techniques were also employed for diagnosis. For example, a study utilized ELISA, flow cytometry, and immunohistochemistry to validate the strong binding of recombinant human anti-αIIbβ3 scFv antibody with platelets. Significant immunoreactivity and affinity binding to platelets were exhibited by the antibodies on both animal and human atheroma plaques (110).

### scFv as therapeutics agent

In addition to their diagnostic values, extensive research on scFv has also applied this technology as a promising approach for the treatment of CVDs. scFv offers new therapeutic avenues to intervene in atherosclerosis and specifically target the pathogenic molecules of the disease, such as LDL, its oxidized forms ox-LDL, malonaldehyde-modified LDL (MDA-LDL), LDL (−), and OxPL. For example, ASA6-scFv showed strong therapeutic potential by specifically binding to Ox-LDL and atherosclerotic plaques. It effectively inhibited the uptake of Ox-LDL by macrophages, which led to a reduction in macrophage apoptosis. As a result, upon administration to ApoE−/− mice, the antibody notably decreased the size of atherosclerotic lesions. Transcriptome analysis indicated that the positive effects were attributed to its impact on fatty acid metabolism and the inhibition of M1 macrophage polarization (11). C2scFv-Crry, a novel construct combining a scFv with a complement inhibitor was also developed to decrease the ox-LDL uptake by macrophages and lower serum ox-LDL levels. These antibody constructs also reduced atherosclerotic plaque formation and C3 deposition in ApoE -/- mice effectively, presenting a promising strategy for treating atherosclerosis by targeting neoepitopes with complement inhibitors (111). Tsimikas et al. demonstrated the significance of scavenger receptor-mediated uptake of Ox-LDL in atherosclerosis and introduced oxidation-specific antibodies, IK17-Fab and IK17-scFv, as effective therapeutic agents. These antibodies inhibited Ox-LDL uptake by macrophages and reduced foam cell formation and atherosclerotic lesion size in LDL receptor knockout mice (112). Moreover, some therapies address other variants of ox-LDL. For instance, a scFvs was engineered to target MDA-LDL in atherosclerotic plaques and used ox-LDL specific Chimeric Antigen Receptors (ox-CARs) by fusing antibodies with components like the IgG4 hinge and CD28/CD3z cytoplasmic domains. These ox-CARs were then incorporated into regulatory T cells (Tregs) via lentiviral transduction. Upon binding to MDA-LDL, ox-CAR-Tregs became activated and exerted anti-inflammatory effects when cultured with human atherosclerotic plaque. Six out of 42 specific CARs designed to target ox-LDL significantly activated Tregs, indicating the potential for engineered CAR-Tregs to reduce inflammation and risk of heart attack and stroke by targeting MDA-LDL in plaques (113). Kazuma et al. developed and expressed 2C7 scFvs in *Pichia pastoris*, with its specific binding to LDL (−) confirmed through ELISA. This antibody inhibited LDL (−) uptake by macrophages, reduced lipid accumulation, downregulated pro-inflammatory gene expression, and significantly reduced atherosclerotic lesions in LDLr-/- mice (96). The nanoformulation technique is also used to target LDL (−) in various studies for therapeutic purposes. For example, Cavalcante et al. developed anti-LDL (−)-MCMN-Zn scFv by using this technique, which showed promising results. Initially, they observed that this nanoformulation was reactive to LDL (−), significantly reduced atherosclerotic lesion area, and downregulated inflammatory markers in LDL receptor knockout (Ldlr-/-) mice, without inducing toxicity (114). Subsequently, they expanded their prior investigation and exhibited that anti-LDL(−)-MCMN-Zn nanoformulation reduces LDL (−) uptake and pro-inflammatory cytokine expression in macrophages. In addition, intravenous administration of this antibody in Ldlr-/- mice effectively inhibited atherosclerosis progression without adverse effects on vascular permeability or leukocyte-endothelium interactions, highlighting its safety and potential therapeutic efficacy (115). Additionally, another type of oxidized lipid, OxPL, was targeted by E06-scFv in different studies. One research demonstrated that transgenic mice overexpressing this antibody showed significantly smaller myocardial infarct sizes compared to control mice after myocardial IR, indicating a potential therapeutic strategy for mitigating atherosclerosis-related myocardial damage (116). Que et al. reported that when the E06-scFv antibody binds with OxPLs, it reduces atherosclerosis and systemic inflammation in transgenic mice, leading to improved cardiovascular health and extended lifespan (97). Concomitantly, researchers also proposed that targeting OxPL with this same E06-scFv can effectively reverse NETosis, atherosclerosis, and arterial thrombosis in LNK-deficient mice (117). Along the same line, the E06-scFv antibody significantly reduced the development of atherosclerosis in IgM-deficient Apoe–/– mice that were fed a low-fat diet. This indicates that E06-scFv has a protective effect against atherosclerosis under these dietary conditions (118).

Since platelets induce thrombosis and play an essential role in atherosclerosis (CVDs), it is also targeted for therapy in different studies. Anti-αIIbβ3 TEG4 scFv, which targets αIIbβ3, the most abundant integrin in platelets, was used for treating atherosclerosis by specifically binding to the platelets within plaques. Grafting these scFv fragments onto nanoparticles enables the precise delivery of therapeutic agents, potentially revolutionizing atherosclerosis treatment (110). Similarly, successful conjugation of a scFv with nanoparticles targeted activated platelets can be achieved by using the staphylococcus aureus sortase A enzyme. This approach preserved the bioactivity of the scFv and enabled strong and specific targeting of scFv-coupled cells and nanoparticles to activated platelets, Unlike the anti-αIIbβ3 scFv, this scFv targets LIBS epitopes on CD41/CD61, which are highly expressed on the surface of activated platelets, offering potential applications in enhancing the efficacy of regenerative cell therapy (42). Likewise, a research team worked on the development of a humanized scFv antibody based on the IV.3 monoclonal antibody that bind to the FcγRIIa receptor on platelets. The humanization was achieved by introducing specific mutations that rendered the molecule human-like but did not affect binding specificity. This scFv antibody targeted platelet factor 4 (PF4) or PF4/heparin complexes and inhibited platelet activation, aggregation, and thrombus formation in heparin-induced thrombocytopenia (HIT) (44). Building upon their prior research, the scientists provided additional data on the efficacy of the humanized scFv in preventing thrombus deposition in an ex vivo microfluidics system. This time the humanization was achieved through a complementarity-determining region grafting and point mutation approach, which aimed at reducing potential immunogenicity for future clinical applications. The study demonstrated that the humanized scFv effectively prevents platelet aggregation and activation induced by HIT immune complexes *in vitro* and stops thrombus deposition *ex vivo* (119). Apart from monospecific antibody, bispecific antibody was also engineered to bind with both activated platelets and peripheral blood mononuclear cells (PBMCs) expressing stem cell antigen-1 (Sca-1), which enhances regenerative cell homing to myocardial damage areas. In a mouse model of ischemia-reperfusion injury, this approach led to successful targeted delivery of PBMCs to the damaged myocardium, reducing inflammatory cell infiltration and improving cardiac function (43). Wang et al. used scFv as a targeting device to deliver a fibrinolytic drug via microbubbles directly to thrombi, ensuring effective treatment without the bleeding risks associated with systemic drugs. This targeted approach allowed for bleeding-free thrombolysis and enhanced the safety and efficacy of thrombotic treatment (99).

In addition, other molecules or cells are targeted by scFv to achieve therapeutic outcomes for CVDs. For example, researchers enhanced the efficacy of LOX-1 targeting scFv by integrating LOX-1-binding peptides, which significantly increased the scFv’s binding activity while maintaining stability. These findings suggest that such modifications could improve the efficacy of LOX-1-targeting scFvs in LOX-1-based therapies for atherosclerotic diseases (107). In another case, by targeting collagen VI alpha 6 chain (COL6A6) with a specific human scFv, researchers achieved a 45% regression in atherosclerosis in ApoE−/− mice, evidenced by aorta staining. This therapy shifted monocytes/macrophages from M1 to M2 phenotypes and modulated cytokines, suggesting its potential for autoimmune-related atherosclerosis (120). A DNA vaccine targeting the CX3CR1 chemokine pathway was developed to address the role of CX3CR1 in mediating monocyte recruitment to atherosclerotic lesions, which is crucial for atherogenesis. Traditional methods to disrupt this pathway have faced translational challenges in human trials, making DNA vaccination a promising alternative that can induce a robust immune response. The DEC-205 (a phagocytosis receptor highly expressed on dendritic cells) single-chain variable fragment was incorporated into the DNA construct to enhance the targeting of dendritic cells, facilitating more effective antigen presentation and immune activation. In this approach, the scFv specific to DEC-205 was not administered as a protein conjugated with nanoparticles but was part of a DNA construct. This construct contained the scFv gene fused to the CX3CR1 gene, allowing targeted delivery and improved dendritic cell uptake. This targeted approach not only increases antibody levels against CX3CR1 but also helps break self-tolerance, leading to significant protection from atherosclerosis by reducing macrophage infiltration and lipid deposition in plaques in the murine model (121). Similarly, VCAM-1-targeted scFv was used in a study, to deliver microRNA-126 directly to the inflamed endothelial cells in the aorta, providing a targeted therapeutic approach to prevent the progression of AAA (98). Moreover, *an in vivo* phage display technique was employed to identify scFvs, that selectively bind to atherosclerotic vascular endothelium and associated tissues, demonstrating their potential as therapeutic agents for targeting atherosclerosis (95).

Generally speaking, as we advance our understanding of CVDs and the role of targeted therapies, scFv stands out as a beacon of hope. Their adaptability and precision position them as a pivotal innovation in theranostic strategies, potentially transforming the landscape of CVD treatment and diagnosis (**Figure 3**) (17).

## Challenges and limitations

Like what has been elucidated above, scFvs are pivotal in theranostic applications and research owing to their superior pharmacokinetic attributes to parental antibodies and their relative ease of cost-effective and large-scale production. Nevertheless, scFvs face several limitations, including issues with specificity, stability, immunogenicity, and scalability (34). Although scFv-based theranostics have demonstrated impressive efficacy in atherosclerosis animal models, they face significant clinical translational hurdles (122).

### Specificity

scFv-mediated theranostic strategies are designed to specifically recognize and bind to specific antigens expressed or exposed in atherosclerotic lesions, such as ox-LDL, VCAM-1, or fibrin (122). However, these antigens may also be present in other tissues or organs, leading to off-target effects or reduced bioavailability (123). Furthermore, the expression of these antigens even on the atherosclerotic tissues may vary depending on the stage and location of the disease. This heterogeneity makes it necessary to arrange multiple scFv ligands for different scenarios to ensure precise targeting (41, 124). Therefore, scFv-based theranostics need to improve their specificity to atherosclerotic plaques by using multiple scFv ligands that target different antigens or epitopes on these lesions (34, 123). Moreover, the genetic engineering of recombinant antibodies has significantly advanced our understanding of the structure and functional organization of immunoglobulins. These advancements have enabled the development of a wide range of engineered antibody molecules for research, diagnosis, and therapy, achieving specificities previously unattainable with conventional antibody technology. Once cloned, the affinity and specificity of antigen binding can be further enhanced by replicating the process of somatic hypermutation that occurs during an immune response. For instance, engineering scFvs with higher affinity for their target antigens can improve molecular specificity. Additionally, modifying the scFv structure to enhance stability and reduce aggregation helps maintain their functional integrity. Techniques such as phage display and affinity maturation can be utilized to select scFvs with optimal binding characteristics. By implementing these approaches, the specificity and overall efficacy of scFv-based therapies can be significantly improved (125–127). Similarly, we can further enhance the specificity and affinity of the already screened antibodies through affinity maturation. *In vivo*, antibody maturation occurs via somatic cell mutation and selective proliferation of specific B-cells under repeated antigen stimulation. *In vitro* antibody maturation replicates the natural process of improving antibody activity by introducing mutations in the V region genes. Various mutation techniques are used to simulate the high-frequency mutations seen *in vivo*. These mutations are then combined with large-scale antibody library construction and screening methods to quickly identify genetically engineered antibodies with higher specificity and affinity, which enhances biological activity (128). Furthermore, the simplest method is to simulate somatic hypermutation through random or point mutations. Other techniques include chain shuffling, DNA shuffling, error-prone PCR, mutagenic *E. coli* mutator cells, and CDR walking. CDR walking iteratively introduces variants exclusively in the CDR regions. Typically, different sub-libraries are prepared and mixed before panning to identify improved mutants. The best antibody from one CDR maturation is then used as the reference for optimizing other CDRs. For example, Steidl et al. achieved a 5,000-fold improvement in affinity by simultaneously diversifying CDR-L3 and CDR-H2 using trinucleotide consensus cassettes, followed by domain combination (128).

### Stability

scFv-based theranostics should maintain their structural integrity and functional activity under such physiological conditions as pH, temperature, proteases, and serum proteins (123). Unfortunately, due to their lack of stabilizing constant domains, scFvs are prone to misfolding, aggregation, thermal instability, and proteolytic degradation, which can reduce their solubility and functionality. They may also face challenges in refolding and can form unwanted dimers or multimers, further impacting their efficacy and production efficiency (41, 129). A promising solution to this problem is the cyclization of scFv antibodies by sortase A-mediated ligation. This methodology decreases open-closed dynamics and aggregation without disrupting antigen affinity or thermal stability (129). Moreover, the stability of scFv molecules may be affected by the physicochemical properties of the nanocarriers attached such as their size, charge, and surface modification (41, 122). To enhance the stability of scFv-based theranostics, various engineering strategies were employed to improve folding, solubility, affinity, and resistance to proteolysis (41, 129). For instance, incorporating disulfide bonds, glycosylation sites, Fc, or fusion partners (such as albumin or PEG) can increase the half-life of scFv molecules (41, 122). Alternatively, scFv-based theranostics can use nanocarriers to protect them from degradation or denaturation in the circulation or at the target site (41). Several research groups have developed methods to prevent scFv aggregation and enhance their stability. Curtis et al. successfully reduced the tendency of scFvs to aggregate by substituting arginine residues, which affect the specificity and selectivity of the antigen-antibody complex, with lysine (130). Although this modification can help design more stable scFvs, it may compromise the specificity and selectivity of antigen-antibody interactions. However, this issue can be mitigated by making mutations in residues that are distant from the binding site (131).

### Immunogenicity

**Figure 4:**
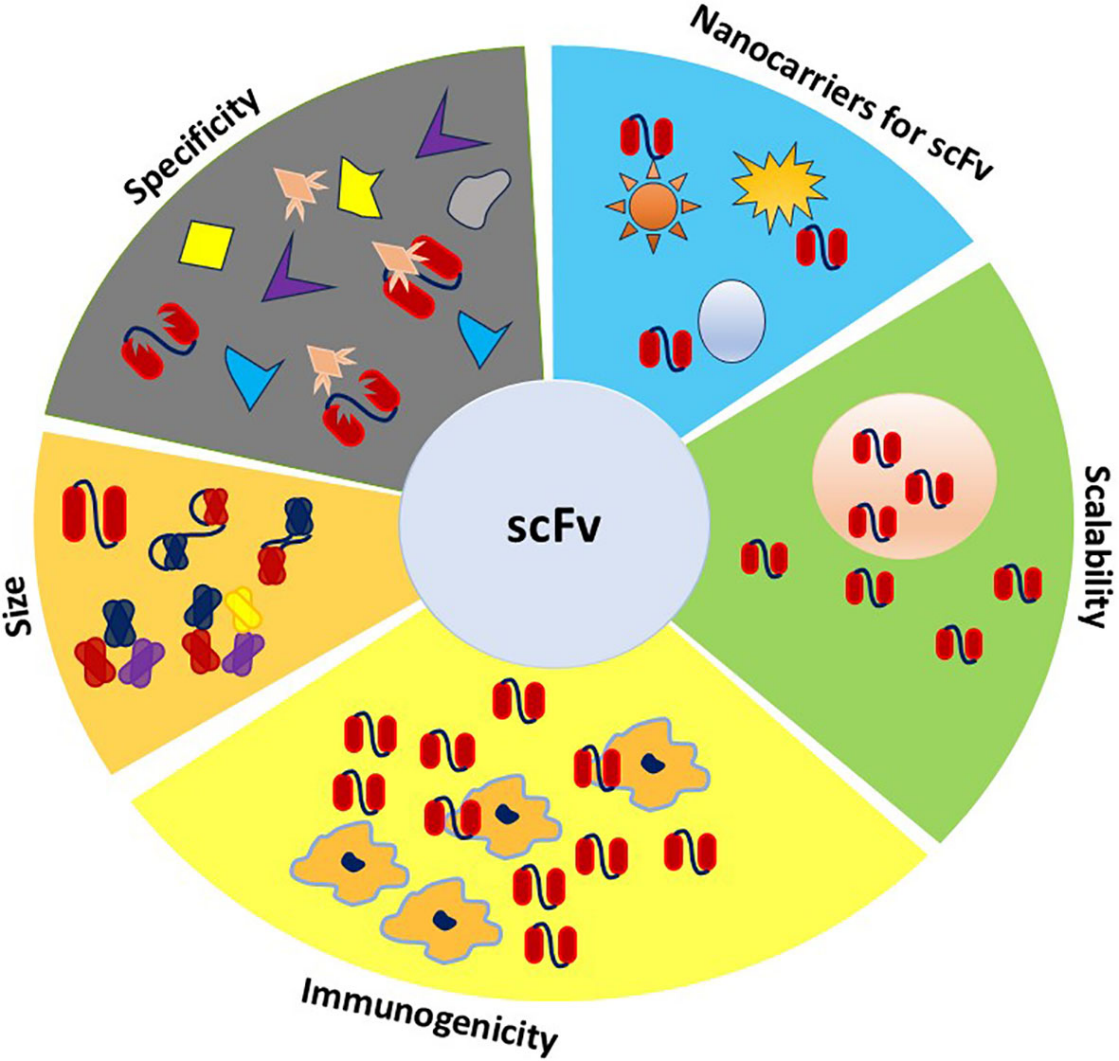
Properties required for ideal theranostic scFv. This figure illustrates the ideal properties of scFvs for theranostic applications, including their versatility in size like bispecific, tri-specific, and tetra-specific configurations, which enable targeting multiple antigens simultaneously. The scFvs specificity ensures precise targeting of disease markers, while low immunogenicity is reflected in the limited recognition by macrophages, reducing the immune response. Scalability is highlighted by their high production capacity, supporting large-scale manufacturing. Additionally, the integration of scFvs with nanoparticles underscores their role in enhancing diagnostic capabilities.

The scFv-derived theranostic proteins need to avoid or minimize the immune responses from the host, such as antibody production, complement activation, phagocytosis (**Figure 4**), or hypersensitivity, because they are foreign proteins, especially if they are derived from non-human sources or contain non-natural amino acids or linkers (41, 122, 132). Despite their smaller size, ease of production, and target specificity, most scFvs face challenges like aggregation and immunogenicity due to their rodent origin, necessitating modifications for human use (41, 122, 133). To make matter worse, scFv molecules may enhance the immunogenicity of third party by cross-linking antigens on the surface of cells or by altering the antigen presentation or processing (41). These immunogenicity issues in theranostic application with scFv can be reduced or overcame by using human or humanized scFv molecules with minimal or no sequence differences from the endogenous antibodies (133). Alternatively, scFv-based theranostics can use stealth nanocarriers that evade immune recognition or clearance by coating with biocompatible or immunomodulatory materials, such as PEG, polysaccharides, and lipids (41).

### Scalability

For effective clinical applications, scFv-mediated theranostics must be produced in large quantities with high purity and quality (122, 134). However, the scFv expression levels are often low and inconsistent (135). Due to their complex structure and low solubility, producing and purifying scFv molecules are challenging tasks (136). To enhance expression levels, various methods can be employed to improve the stability of the generated fragments, including the modification of specific residues within the structure and the application of random mutagenesis (137). Bacterial expression systems like *E. coli* are beneficial for generating scFvs in high yields compared to full-sized antibodies, and various host strains, particularly *E. coli*, have been used to produce recombinant antibody fragments by directing their secretion into the periplasmic space. Unfortunately, producing large amounts of scFvs in bacteria often results in insoluble inclusion bodies and aggregates (136). Some researchers have also proposed humanizing scFvs by substituting hydrophobic amino acids with hydrophilic ones to reduce the likelihood of aggregation (138). Interestingly, alternative production methods, like yeast, plant, or mammalian expression systems, can be utilized for scFv-mediated theranostic strategies (134, 135). Mammalian cells possess advanced protein folding and glycosylation systems within the endoplasmic reticulum, enabling the production of antibodies that closely resemble hybridoma antibodies in structure and activity. Consequently, they are commonly used for expressing whole antibody molecules. Additionally, insect expression systems share some benefits with mammalian cells, such as efficient protein folding and glycosylation. They are also less expensive to produce, more efficient in expression, and more suitable for large-scale production compared to mammalian expression systems (134, 135). Plant expression systems for producing single-chain variable fragments (scFvs) have gained significant attention due to their scalability, cost-effectiveness, and ability to perform post-translational modifications. Studies have shown that transgenic tobacco and rice can effectively produce functional scFvs, with tobacco allowing secretion into the apoplast and rice offering low allergenicity, while other cereal crops like wheat provide additional benefits for protein accumulation and storage (139). Alternatively, the yeast system offers a combination of advantages from both E. coli and mammalian cells. Its genetic system is more advanced than that of E. coli and easier to manipulate than that of mammalian cells. For instance, the expression system *Pichia pastoris* addresses these challenges by achieving high yields, retaining strong biological activity, and secreting recombinant proteins directly into the supernatant. This method avoids common issues associated with bacterial cytoplasmic production, such as low yield, poor solubility, and reduced affinity (110). Nevertheless, extensive characterization and validation are necessary to ensure the specificity, stability, immunogenicity, and bioactivity of scFv molecules (41, 122, 124).

## Future perspectives and conclusion

Over the past decades, significant progress has been made in the development of novel targeting antibody molecules. The technologies used to synthesize these molecules have continuously evolved, addressing constraints related to purification, safety, stability, circulation half-life, efficacy, immunogenicity, and adverse effects in clinical applications. Despite their limitations, scFv antibodies have been utilized for various purposes, including treatment and diagnosis. scFv has unique advantages and great potential, in providing new ideas and approaches to diagnose and treat CVDs. In addition to exploring known atherosclerosis-related epitopes, it is essential to continue identifying new therapeutic targets. For instance, the research group led by Almudena R. Ramiro at the Spanish National Centre for Cardiovascular Research (CNIC) conducted high-throughput single-cell analyses of antibody libraries related to atherosclerosis. By sequencing antibody genes from over 1,700 B cells derived from atherosclerotic Ldlr-/- and control mice, they identified 56 antibodies expressed by *in vivo* expanded B lymphocyte clones associated with atherosclerosis. Remarkably, one-third of these amplified antibodies showed reactivity to atherosclerotic plaques, indicating that various antigens within the lesion can elicit antibody responses. Further in-depth proteomic analysis revealed that ALDH4A1, a mitochondrial dehydrogenase involved in proline metabolism, is the target antigen of one such autoantibody, A12 (140). Compared with traditional drug delivery methods, scFv targets different ligands according to different pathological mechanisms, which can cover such pathogenic targets as MMPs that lead to plaque rupture (141), CD36 that facilitates ox-LDL uptake and foam cell formation (142), annexin A5 that marks plaque vulnerability (143), and FGL2 that triggers thrombosis (144). Building on the exploration of new therapeutic targets for atherosclerosis in the future, another promising approach is the use of BiTE (bispecific T cell engager) therapy. This innovative treatment combines two scFv from different monoclonal antibodies through a short and flexible linker. This design allows the construct to freely rotate and fold, facilitating interactions with target receptors on the membranes of two distinct epitopes or cell surface targets. For instance, BiTE can target an MDA-LDL-related antigen and regulatory T cells (Tregs), thereby inhibiting the progression of atherosclerosis (68). Furthermore, scFv-based ultrasound and photoacoustic imaging techniques provided real-time and high-resolution views of atherosclerotic plaques, while scFv-based PET scans offered detailed quantitative analyses. In addition, cyclizing scFvs improved the antibody’s stability and affinity. Moreover, nanobubbles and nano-emulsions containing scFv proved to be adaptable carriers for both imaging and therapeutic agents. In addition, a recently developed practice known as thermotherapy involves binding small antibody fragments to magnetic nanoparticles (MNPs). These MNPs can selectively target atherosclerosis-related antigens. This approach holds promise for the future as an effective tool in bio-nano-based targeting technologies for the early detection of atherosclerosis. The scFv antibody fragments, due to their inherent miniaturization, are more flexible and can be enhanced to address issues related to full-size monoclonal antibodies in the treatment and diagnosis of solid tissues. Unlike whole antibody molecules, which are generally confined to the extracellular environment, scFv antibodies can be conjugated with nanocarriers, cell-penetrating peptides, or sequences that enable them to recognize and bind to cell surface receptors, facilitating their internalization into target cells via receptor-mediated endocytosis.

This review has covered an overview that scFv antibodies offer unique advantages and significant potential in diagnosing and treating cardiovascular diseases (CVDs). Their flexibility and miniaturized structure allow for innovative approaches that address limitations associated with full-size monoclonal antibodies. By leveraging these properties, scFv antibodies can enhance the precision and effectiveness of therapeutic and diagnostic strategies, paving the way for new advancements in CVD management. scFv molecules are revolutionizing cardiovascular theranostic by offering high-affinity antigen targeting, future integration with advanced technologies and novel delivery systems will mitigate challenges this modern strategy brought in specificity, stability, immunogenicity, and scalability (**Figure 4**). For example, applying scFvs in regenerative medicine may improve stem cell therapy efficacy, and leveraging scFv technology for individual solutions can advance personalized medicine. Ongoing advancements in biotechnology are poised to significantly improve the functionality and clinical application of scFvs, establishing them as a cornerstone in the management of CVD. Ultimately, the successful integration of scFv-based theranostics into clinical settings has great potential to transform the landscape of CVD care, leading to more precise and personalized diagnosis, as well as effective treatment strategies for patients.
